# Evaluating the performance of ChatGPT-3.5 and ChatGPT-4 on the Taiwan plastic surgery board examination

**DOI:** 10.1016/j.heliyon.2024.e34851

**Published:** 2024-07-18

**Authors:** Ching-Hua Hsieh, Hsiao-Yun Hsieh, Hui-Ping Lin

**Affiliations:** Department of Plastic Surgery, Kaohsiung Chang Gung Memorial Hospital, Chang Gung University and College of Medicine, Kaohsiung, 83301, Taiwan

**Keywords:** ChatGPT-3.5, ChatGPT-4, Taiwan plastic surgery board examination

## Abstract

**Background:**

Chat Generative Pre-Trained Transformer (ChatGPT) is a state-of-the-art large language model that has been evaluated across various medical fields, with mixed performance on licensing examinations. This study aimed to assess the performance of ChatGPT-3.5 and ChatGPT-4 in answering questions from the Taiwan Plastic Surgery Board Examination.

**Methods:**

The study evaluated the performance of ChatGPT-3.5 and ChatGPT-4 on 1375 questions from the past 8 years of the Taiwan Plastic Surgery Board Examination, including 985 single-choice and 390 multiple-choice questions. We obtained the responses between June and July 2023, launching a new chat session for each question to eliminate memory retention bias.

**Results:**

Overall, ChatGPT-4 outperformed ChatGPT-3.5, achieving a 59 % correct answer rate compared to 41 % for ChatGPT-3.5. ChatGPT-4 passed five out of eight yearly exams, whereas ChatGPT-3.5 failed all. On single-choice questions, ChatGPT-4 scored 66 % correct, compared to 48 % for ChatGPT-3.5. On multiple-choice, ChatGPT-4 achieved a 43 % correct rate, nearly double the 23 % of ChatGPT-3.5.

**Conclusion:**

As ChatGPT evolves, its performance on the Taiwan Plastic Surgery Board Examination is expected to improve further. The study suggests potential reforms, such as incorporating more problem-based scenarios, leveraging ChatGPT to refine exam questions, and integrating AI-assisted learning into candidate preparation. These advancements could enhance the assessment of candidates' critical thinking and problem-solving abilities in the field of plastic surgery.

## 1 Introduction

Chat Generative Pre-Trained Transformer (ChatGPT) developed by OpenAI is a state-of-the-art language model that has been tested across various domains. GPT-3.5, a variant of GPT-3, was introduced to utilize the transformer architecture but with enhancements that increased its understanding and generation capabilities. GPT-3.5 exhibited better context understanding and more nuanced text generation compared to its predecessor, making it more effective in various tasks like conversation, content creation, and even coding [1,2]. In addition, with more parameters than GPT-3.5, GPT-4 showed notable improvements in reasoning, understanding context, and generating more coherent and contextually relevant responses. It also reduced the generation of incorrect or nonsensical information, a challenge in earlier versions [3]. Its performance provides insight into its potential application in medical practice and education [[4], [5], [6], [7]].

ChatGPT has been evaluated in a variety of medical fields, including medical license examinations, where a portion of the programs passed and others did not. For example, ChatGPT-4 achieved an accuracy of 86 % on the Peruvian National Licensing Medical Examination, outperforming GPT-3.5 and most examinees [8]. In the United States Medical Licensing Examination (USMLE), ChatGPT-4 answered 88 % of Step 1 questions correctly and 90 % of Step 3 questions correctly [9]. ChatGPT-4 reached the passing standard for the National Medical Licensing Examination in Japan with an 81.5 % correct response rate [10]. On the Brazilian National Examination for Medical Degree Revalidation, ChatGPT-4 answered 87.7 % of questions correctly [11]. In contrast, ChatGPT has been failed in some medical licensing examinations. ChatGPT did not pass the threshold score in the Chinese National Medical Licensing Examination, with the highest recorded score being 54.67 % in some years [12]. In the Taiwanese Pharmacist Licensing Examination, ChatGPT-3.5 failed, with a correct rate of 54.4 % and 56.9 % in different stages [13]. ChatGPT performed poorly in the Nephrology Test Questions with an accuracy rate of 51 % for GPT-3.5 and 74 % for GPT-4, which is below the passing threshold [14]. ChatGPT did not pass the medical specialty exam in a study observing its ranking among the candidates over the last five years [15].

The Taiwan Plastic Surgery Board Examination is a test for surgeons who wish to specialize in plastic surgery. The examination is designed to test the knowledge and skills of medical professionals in the field of plastic surgery and to ensure that they meet the standards required for certification as a plastic surgery specialist in Taiwan. The examination is conducted by the Taiwan Plastic Surgery Society and is divided into two parts: written and oral. The written examination consists of multiple-choice questions or single-choice question in Chinese, with some technical terms allowed to be written in English. Some questions would consist of graphs and tables. The content of the written examination covers basic principles of surgery and related basic medical knowledge, as well as specialized knowledge in fields such as orthopedic surgery, urology, plastic surgery, cardiovascular surgery, neurology, anesthesiology, pediatric surgery, thoracic and abdominal surgery, and colorectal surgery. All questions of the written examination should be established based on the textbook of 4th edition of Plastic Surgery edited by Peter Neligan, which book is 6-vol set with 4680 pages [16]. The time allotted for the written examination is 2 h. Furthermore, the oral examination is conducted by a panel of five oral examiners and covers topics such as the diagnosis and treatment of surgical diseases, as well as related anatomy, bacteriology, serology, physiology, pathology, anesthesiology, and nuclear medicine. The examination is graded on a percentage basis, with the written examination requiring a score of 60 to pass and the oral examination requiring an average score of 60 from the nine oral examiners to pass. For those who participate in the academic convention of the Taiwan Society of Plastic Surgery and obtain certified documents, each time they participate, the score of the written examination of the previous specialist physician is added to one point, but not more than five points in each examination. The examination is held annually, and the number of applicants would be around dozens.

In Taiwan, to pass the Taiwan Plastic Surgery Board Examination is difficult, as candidates must complete tough and diverse tests in a limited amount of time. The objective of this research was to evaluate the efficacy of ChatGPT-3.5 and ChatGPT-4 in answering questions derived from official examinations conducted in the last eight years.

## 2 Ethical consideration

This study did not include any human participants or patient information. The data utilized in this study are accessible to the public on the Internet. Consequently, the study was deemed ineligible for review by the hospital's Institutional Review Board.

## 3 Methods

In this study, we evaluated the large language model (LLM) models ChatGPT-3.5 (which is truly the ChatGPT-3.5-turbo) and GPT-4 produced by OpenAI (https://openai.com). The performance data of the plastic surgeon board examinations done using ChatGPT-3.5 and ChatGPT-4 for eight years of official tests with a total of 1375 questions, which included 985 single-choice tests and 390 multiple-choice tests. No questions were discarded, even if they contained images, tables, or charts that ChatGPT-3.5 did not recognize but ChatGPT-4 did. The responses to ChatGPT-3.5 and GPT-4 were obtained between June and July 2023. Two assistants manually inserted the questions into the text input field in the sequence of examination, using the prompt "This is the single-choice/multiple-choice question, please select the correct answer". If the model only provided one answer for the multiple-choice questions, or several responses for the single-choice questions, the question was re-entered to select the proper number(s) of responses to the questions. In this study, the user profile for ChatGPT-4 did not specify being a plastic surgeon or related profession. To eliminate any memory retention bias, a new chat session was launched for each question. Memory retention with recurrent neural networks can happen when the large language model learns new information and then applies it to future data inputs and outputs.

## 4 Results

### 4.1 Overall performance

As demonstrated in Table 1, Table 2, the data illustrate ChatGPT's performance in answering questions on plastic surgeon board exams throughout an 8-year period. The overall number of examinations taken has increased from 140 in 2015 to 243 in 2022, showing that the exam is being expanded. When comparing the two ChatGPT versions, the performance improvement from ChatGPT-3.5 to ChatGPT-4 is quite substantial. ChatGPT-3.5 had an overall correct answer rate of 41 % across the 8-year period, while ChatGPT-4 achieved a significantly higher 59 % correct rate. This represents almost a 20 percentage point increase in overall accuracy between the two model versions. ChatGPT-4 successfully passed five out of eight yearly exams, but ChatGPT-3.5 failed in all of the tests throughout all years, considering the criteria for a successful pass being established at 60 % accuracy without taking into account the inclusion of additional points.

**Table 1:** Performance of ChatGPT-3.5 in answering the questions of eight years of official tests.

ChatGPT 3.5										
Years	Performance of the answering to the test of each year				Performance of the answering to single-choice questions			Performance of the answering to multiple-choice questions		
	Total number of tests	Number of correct answers	Number of incorrect answers	AI Answer Correct Rate	Total number of tests	Number of correct answers	AI Answer Correct Rate	Total number of tests	Number of correct answers	AI Answer Correct Rate
2015	140	53	87	38 %	98	47	48 %	42	6	14 %
2016	150	77	73	51 %	108	66	61 %	42	11	26 %
2017	130	71	59	55 %	94	60	64 %	36	11	31 %
2018	160	67	93	42 %	111	53	48 %	49	14	29 %
2019	150	58	92	39 %	108	45	42 %	42	13	31 %
2020	160	46	114	29 %	107	39	36 %	53	7	13 %
2021	242	92	150	38 %	179	76	42 %	63	16	25 %
2022	243	96	147	40 %	180	83	46 %	63	13	21 %
Total	1375	560	815	41 %	985	469	48 %	390	91	23 %

**Table 2:** Performance of ChatGPT-4 in answering the questions of eight years of official tests.

ChatGPT 4.0										
Years	Performance of the answering to the test of each year				Performance of the answering to single-choice questions			Performance of the answering to multiple-choice questions		
	Total number of tests	Number of correct answers	Number of incorrect answers	AI Answer Correct Rate	Total number of tests	Number of correct answers	AI Answer Correct Rate	Total number of tests	Number of correct answers	AI Answer Correct Rate
2015	140	63	77	45 %	98	52	53 %	42	11	26 %
2016	150	92	58	61 %	108	73	68 %	42	19	45 %
2017	130	89	41	68 %	94	73	78 %	36	16	44 %
2018	160	102	58	64 %	111	76	68 %	49	26	53 %
2019	150	88	62	59 %	108	65	60 %	42	23	55 %
2020	160	91	69	57 %	107	69	64 %	53	22	42 %
2021	242	147	95	61 %	179	118	66 %	63	29	46 %
2022	243	146	97	60 %	180	123	68 %	63	23	37 %
Total	1375	818	557	59 %	985	649	66 %	390	169	43 %

### 4.2 Yearly trend

The accuracy of ChatGPT-3.5 varied between 38 % and 55 %, whereas ChatGPT-4 had a range of 45 %–68 %. The annual performance trends likewise demonstrate a distinct superiority for ChatGPT-4. Consistently, ChatGPT-4 surpassed the ChatGPT-3.5 in performance, frequently by significant margins. In 2011, there was a remarkable disparity in accuracy between ChatGPT and ChatGPT-4, with ChatGPT achieving a 38 % accuracy rate, while ChatGPT-4 reached 61 % accuracy, resulting in a substantial 23 % difference.

### 4.3 Question type performance

When it came to single-choice questions, both ChatGPT versions outperformed the multiple-choice ones. But compared to ChatGPT-3.5, ChatGPT-4 performed noticeably better on both single- and multiple-choice questions. ChatGPT-4 outperformed ChatGPT-3.5 on the single-choice problems, increasing from 48 % to 66 % right. Using a 60 % accuracy level, ChatGPT-3.5 could pass two of the eight-year single-choice tests, whereas ChatGPT-4 could pass seven of the eight-year tests. With regard to the multiple-choice questions, GhatGPT-4 outperformed ChatGPT-3.5, essentially doubling the performance, with an accurate rate of 43 % compared to ChatGPT-3.5's 23 %. This suggests that the ChatGPT-4 has advanced significantly in its ability to handle open-ended, more complicated question kinds.

## 5 Discussion

This analysis found that GhatGPT-4 outperformed ChatGPT-3.5 in the Taiwan Plastic Surgery Board Examination, passing the majority of exams during an eight-year period. As the future iterations of ChatGPT evolve in the near future, we should expect the considerable improvements in contextual knowledge, reasoning, and multimodal interactions, increasing the pass rate on this exam. These versions also likely offer more nuanced simulation and problem-solving exercises and enhances capabilities in generating and validating examination questions.

ChatGPT-4 exhibits a substantial enhancement over ChatGPT-3.5 in a variety of domains, such as the capacity to assess medical knowledge [17], the processing of complicated clinical information [18], and the management of imaging [19]. Furthermore, GPT-4 is highly proficient in the comprehension and reasoning of natural language, which results in significant improvements in natural language processing tasks [20]. ChatGPT-4 consistently exhibited superior accuracy and proficiency, particularly in the context of challenging and specific disease questions, surpassing ChatGPT-3.5 [21]. Therefore, it is unsurprising that ChatGPT-4 outperformed ChatGPT-3.5 in a variety of tasks and domains, including the generation of accurate International Classification of Diseases (ICD) billing codes [22], the answering of ophthalmology questions [[23], [24], [25]], and the passing of the board examinations for urology [26], dentist [27], emergency medicine [28], and medical licensing [29]. Clearly, ChatGPT-4's capacity to manage pictures in the test provides a greater advantage than ChatGPT-3.5 regarding the likelihood of passing the examination in this study. These results collectively indicate that ChatGPT-4 demonstrates superior performance in a variety of tasks and specialties when contrasted with ChatGPT-3.5, highlighting advancements in language model capabilities and potential for enhanced applications in various fields.

Further, we attempted to respond to one issue about the emergence of artificial intelligence (AI).

“When AI can simply pass a test, does that detract the value of the test?”

When AI can pass a test designed for humans, it prompts a reevaluation of the test's objectives and value. While AI's success, such as ChatGPT passing medical licensing exams, demonstrates its growing cognitive capabilities, it also underscores the need to ensure tests assess uniquely human attributes like ethical reasoning, empathy, and practical judgment. Rather than detracting from the test's value, AI's achievements can encourage the redesign of assessments to better capture the nuanced and complex nature of human intelligence and professional competence [30,31]. Thus, AI's ability to pass tests should not diminish their value but rather drive innovation in how we define and measure expertise, ensuring that assessments remain relevant in distinguishing human proficiency in an era of advanced AI [30,31]. These advancements underscore the potential of AI to not only transform how medical knowledge is assessed but also to enhance the quality of exanimation.

Moreover, the use of AI in critical medical assessments gives rise to substantial ethical considerations. While AI's performance demonstrates its potential in medical education, it challenges our understanding of medical competence and expertise. There are risks of over-reliance on AI, potentially undermining critical thinking skills essential for medical professionals. Concerns about cheating and exam integrity also arise. Additionally, AI use could exacerbate educational inequalities. As AI capabilities grow, we must reconsider how to assess uniquely human skills like empathy and ethical reasoning in medical practice. Balancing AI's benefits in medical education with these ethical considerations is crucial for maintaining the integrity and effectiveness of medical assessments.

Based on the results and insights gained from the study regarding the Taiwan Plastic Surgery Board Examination, here are some suggestions for potential reforms. First, one key area for improvement is the examination format and question types. Currently, the exams seem to rely heavily on memorization-based questions, which may not adequately assess the critical thinking and problem-solving abilities required of practicing plastic surgeons. A shift towards more "Problem-Based Learning" (PBL) style questions, where candidates are presented with clinical scenarios and asked to analyze, diagnose, and propose treatment plans, could better evaluate the applicants' real-world competencies.

Second, the exam board could innovate by leveraging ChatGPT's capabilities to pre-test the exam questions and identify any issues with clarity, ambiguity, or difficulty level before administering the actual exam. For example, ChatGPT discovered an error in one test stating the most prevalent gene translocation of anaplastic lymphoma kinase in breast implant-associated anaplastic large cell lymphoma, which is implicated in chromosomal 2q23, but the correct response is 2p23. This could help the exam board refine the questions, ensure a fair assessment, and set a reasonable threshold for passing. The Taiwan Plastic Surgery Board Examination has set questions only based on the textbooks, making the textbooks the only way to determine which is true or false in the plastic surgery area. Using the ChatGPT to pre-check the questions may avoid confusion or ambiguous opinions between the textbook and the vast literature on plastic surgery.

Third, students can use the AI to take mock tests, clarify hard topics, and receive rapid feedback on their responses. This collaborative interaction may improve learning, retention, and utilization of medical information. The access to ChatGPT or similar AI tools may enable exam candidates to better prepare for the exam. Applicants could use these tools to practice answering various types of questions, organize their knowledge, and recommend topics for additional study. Integrating AI-assisted learning into exam preparation can greatly improve applicants' readiness and performance.

Fourth, the Taiwan Plastic Surgery Board Examination set difficult questions from one set of textbooks with the intention of differentiating the levels of candidates. Therefore, certain questions rely on unrealistic memories, which are not important in clinical thinking. Given the expectation for ChatGPT to pass all tests in the near future, it might be beneficial to modify the test's spirit to evaluate eligible candidates. This could involve establishing a comprehensive question bank that encompasses the entire specialty, rather than scrutinizing the candidate with impractical and challenging questions. The spirit of the test should be adjusted to qualify the candidates as plastic surgeons rather than give them a passing order.

At last, future research should focus on developing AI-enhanced problem-based learning scenarios and comparing AI performance with human medical professionals across specialties. Investigating how AI can help create more comprehensive assessment methods that evaluate both knowledge and critical thinking is crucial. Studies on using AI to personalize medical education and identify knowledge gaps are beneficial. Furthermore, longitudinal studies on the effects of AI integration in medical curricula on student performance and patient outcomes would provide essential data for shaping future medical education policies.

This study has several limitations. It focuses solely on the Taiwan Plastic Surgery Board Examination, potentially limiting generalizability to other medical exams. The absence of human performance comparisons makes it difficult to contextualize the AI's results. Potential biases in question selection and language nuances were not addressed. While there is difference of performance in answering single-choice and multiple-choice questions, what types of questions posed more challenges are difficult to analyze, seeing the analysis focuses on correct answer rates without evaluating the quality of AI explanations. Each question was only tested once, not accounting for potential variability in AI responses. The study only assesses written questions, omitting practical clinical components crucial for medical competency. Given the rapid pace of AI development, these results may quickly become outdated. Lastly, the study does not explore how different prompting strategies might affect AI performance, limiting our understanding of the AI's full capabilities in this context.

## 6 Conclusion

This study revealed that ChatGPT-4 significantly outperformed its predecessor, ChatGPT-3.5, in answering questions from the Taiwan Plastic Surgery Board Examination. The superior performance of GPT-4 was particularly notable in both single-choice and multiple-choice question types. As ChatGPT continues to evolve, we can expect further improvements in passing the examination in the near future. This raises important questions about the future design and purpose of such assessments, and how they can be reformed to better evaluate the nuanced skills and judgment required of a plastic surgeon. Leveraging the capabilities of advanced language models like ChatGPT may offer new opportunities to enhance the quality and fairness of medical licensing examinations.

## Funding

This research was supported by a grant from CORPG8N0471.

## CRediT authorship contribution statement

**Ching-Hua Hsieh:** Writing – original draft, Funding acquisition, Conceptualization. **Hsiao-Yun Hsieh:** Resources. **Hui-Ping Lin:** Resources.

## Declaration of competing interest

The authors declare the following financial interests/personal relationships which may be considered as potential competing interests:Ching-Hua Hsieh reports a relationship with Kaohsiung Chang Gung Memorial Hospital that includes: funding grants. If there are other authors, they declare that they have no known competing financial interests or personal relationships that could have appeared to influence the work reported in this paper.

## References

[1] Talebi S., Tong E., Mofrad M.R.K. (2023).

[2] Bayani D. (2023). GPT3.5's Abilities in Regard to Recognizing and Generating ASCII-Art Are Not Totally Lacking.

[3] OpenAI J. Achiam, Adler S., Agarwal S., Ahmad L., Akkaya I. (2023).

[4] Mu Y., He D. (2024). The potential applications and challenges of ChatGPT in the medical field. Int. J. Gen. Med..

[5] Xu X., Chen Y., Miao J. (2024). Opportunities, challenges, and future directions of large language models, including ChatGPT in medical education: a systematic scoping review. J Educ Eval Health Prof.

[6] Tan S., Xin X., Wu D. (2024). ChatGPT in medicine: prospects and challenges: a review article. Int. J. Surg..

[7] Benítez T.M., Xu Y., Boudreau J.D., Kow A.W.C., Bello F., Van Phuoc L., Wang X., Sun X., Leung G.K., Lan Y., Wang Y., Cheng D., Tham Y.C., Wong T.Y., Chung K.C. (2024). Harnessing the potential of large language models in medical education: promise and pitfalls. J. Am. Med. Inf. Assoc..

[8] Flores-Cohaila J.A., García-Vicente A., Vizcarra-Jiménez S.F., De la Cruz-Galán J.P., Gutiérrez-Arratia J.D., Quiroga Torres B.G., Taype-Rondan A. (2023). Performance of ChatGPT on the Peruvian national licensing medical examination: cross-sectional study. JMIR Med Educ.

[9] Mihalache A., Huang R.S., Popovic M.M., Muni R.H. (2024). ChatGPT-4: an assessment of an upgraded artificial intelligence chatbot in the United States Medical Licensing Examination. Med. Teach..

[10] Yanagita Y., Yokokawa D., Uchida S., Tawara J., Ikusaka M. (2023). Accuracy of ChatGPT on medical questions in the national medical licensing examination in Japan: evaluation study. JMIR Form Res.

[11] Gobira M., Nakayama L.F., Moreira R., Andrade E., Regatieri C.V.S., Belfort R. (2023). Performance of ChatGPT-4 in answering questions from the Brazilian national examination for medical degree revalidation. Rev. Assoc. Med. Bras..

[12] Zong H., Li J., Wu E., Wu R., Lu J., Shen B. (2024). Performance of ChatGPT on Chinese national medical licensing examinations: a five-year examination evaluation study for physicians, pharmacists and nurses. BMC Med. Educ..

[13] Wang Y.-M., Shen H.-W., Chen T.-J. (2023). Performance of ChatGPT on the pharmacist licensing examination in Taiwan. J. Chin. Med. Assoc..

[14] Miao J., Thongprayoon C., Garcia Valencia O.A., Krisanapan P., Sheikh M.S., Davis P.W., Mekraksakit P., Suarez M.G., Craici I.M., Cheungpasitporn W. (2024). Performance of ChatGPT on Nephrology test questions. Clin. J. Am. Soc. Nephrol..

[15] Oztermeli A.D., Oztermeli A. (2023). ChatGPT performance in the medical specialty exam: an observational study. Medicine.

[16] Neligan P.C. (2017).

[17] Lingjiao Chen M.Z., Zou James (2023). How is ChatGPT's behavior changing over time?. arXiv.

[18] Oh N., Choi G.S., Lee W.Y. (2023). ChatGPT goes to the operating room: evaluating GPT-4 performance and its potential in surgical education and training in the era of large language models. Ann Surg Treat Res.

[19] Cheng K., Sun Z., He Y., Gu S., Wu H. (2023). The potential impact of ChatGPT/GPT-4 on surgery: will it topple the profession of surgeons?. Int. J. Surg..

[20] Rahaman M.S., Ahsan M.M.T., Anjum N., Terano H.J.R., Rahman M.M. (2023). From ChatGPT-3 to GPT-4: a significant advancement in AI-driven NLP tools. Journal of Engineering and Emerging Technologies.

[21] Takagi S., Watari T., Erabi A., Sakaguchi K. (2023). Performance of GPT-3.5 and GPT-4 on the Japanese medical licensing examination: comparison study, JMIR. Med. Educ..

[22] Amani S., Benjamin S., Glicksberg, Eyal Zimlichman, Yiftach Barash, Robert Freeman, Alexendar, Charney G., Nadkarni, Eyal Klang (2023). Assessing GPT-3.5 and GPT-4 in generating international classification of diseases billing codes. medRxiv.

[23] Xue X., Zhang D., Sun C., Shi Y., Wang R., Tan T., Gao P., Fan S., Zhai G., Hu M., Wu Y. (2024). Xiaoqing: a Q&A model for glaucoma based on LLMs. Comput. Biol. Med..

[24] Wong M., Lim Z.W., Pushpanathan K., Cheung C.Y., Wang Y.X., Chen D., Tham Y.C. (2023). Review of emerging trends and projection of future developments in large language models research in ophthalmology. Br. J. Ophthalmol..

[25] Cheong K.X., Zhang C., Tan T.E., Fenner B.J., Wong W.M., Teo K.Y., Wang Y.X., Sivaprasad S., Keane P.A., Lee C.S., Lee A.Y., Cheung C.M.G., Wong T.Y., Cheong Y.G., Song S.J., Tham Y.C. (2024). Comparing generative and retrieval-based chatbots in answering patient questions regarding age-related macular degeneration and diabetic retinopathy. Br. J. Ophthalmol..

[26] Tsai C.Y., Hsieh S.J., Huang H.H., Deng J.H., Huang Y.Y., Cheng P.Y. (2024). Performance of ChatGPT on the Taiwan urology board examination: insights into current strengths and shortcomings. World J. Urol..

[27] Ohta K., Ohta S. (2023). The performance of GPT-3.5, GPT-4, and bard on the Japanese national dentist examination: a comparison study. Cureus.

[28] Lee G.U., Hong D.Y., Kim S.Y., Kim J.W., Lee Y.H., Park S.O., Lee K.R. (2024). Comparison of the problem-solving performance of ChatGPT-3.5, ChatGPT-4, Bing Chat, and Bard for the Korean emergency medicine board examination question bank. Medicine (Baltim.).

[29] Rojas M., Rojas M., Burgess V., Toro-Pérez J., Salehi S. (2024).

[30] Fan M., Yang X., Yu T., Liao Q.V., Zhao J. (2022). Human-AI collaboration for UX evaluation: effects of explanation and synchronization. Proc. ACM Hum.-Comput. Interact..

[31] Jutel M., Zemelka-Wiacek M., Ordak M., Pfaar O., Eiwegger T., Rechenmacher M., Akdis C.A. (2023). The artificial intelligence (AI) revolution: how important for scientific work and its reliable sharing. Allergy.

